# Genetic and Dietary Determinants of Insulin-Like Growth Factor (IGF)-1 and IGF Binding Protein (BP)-3 Levels among Chinese Women

**DOI:** 10.1371/journal.pone.0108934

**Published:** 2014-10-06

**Authors:** Qiong Wang, Li Liu, Hui Li, Lauren E. McCullough, Ya-na Qi, Jia-yuan Li, Jing Zhang, Erline Miller, Chun-xia Yang, Jennifer S. Smith

**Affiliations:** 1 Department of Epidemiology and Biostatistics, West China School of Public Health, Sichuan University, Chengdu, P.R. China; 2 The Comprehensive Guidance Center of Women's Health, Chengdu Women's and Children's Central Hospital, Chengdu, P.R. China; 3 Department of Epidemiology, Gillings School of Global Public Health, University of North Carolina, Chapel Hill, North Carolina, United States of America; 4 Lineberger Comprehensive Cancer Center, Chapel Hill, North Carolina, United States of America; 5 Department of Health Service Management, Public Health School, Sun Yat-Sen University, Guangzhou, Guangdong, China; Duke University Medical Center, United States of America

## Abstract

**Background:**

Higher insulin-like growth factor (IGF)-1 and lower IGF binding protein (BP)-3 levels have been associated with higher commoncancer risk, including breast cancer. Dietary factors, genetic polymorphisms, and the combination of both may influence circulating IGF-1 and IGFBP-3 serum concentrations.

**Methods:**

From September 2011 to July 2012, we collected demographic, reproductive and dietary data on 143 women (≥40 years). We genotyped *IGF-1* rs1520220 and *IGFBP-3* rs2854744 and measured circulating IGF-1 and IGFBP-3 levels in serum. Covariance analyses were used to estimate the associations of serum levels of IGF-1 and IGFBP-3, and the molar ratio of IGF-1to IGFBP-3 with *IGF-1* rs1520220 and *IGFBP-3* rs2854744 genotypes. We subsequently assessed the combined influence of genetics and diet (daily intake of protein, fat and soy isoflavones) on IGF-1 and IGFBP-3 levels.

**Results:**

Among women aged less than 50 years, circulating IGF-1 serum levels were significantly lower for those with CC genotype for *IGF-1* rs1520220 than levels for those with the GC or GG genotypes (in recessive model: *P* = 0.007).In gene-diet analyses among these women, we found carrying CC genotype for *IGF-1* rs1520220 and high soy isoflavone intake tend to be associated with lower circulating IGF-1 levels synthetically (*P* = 0.002). Women with GG or GC genotypes for *IGF-1* rs1520220 and with low intake of soy isoflavones had the highest levels of circulating IGF-1 (geometric mean [95% CI]: 195 [37, 1021] µg/L). Comparatively, women with both the CC genotype and high soy intake had the lowest levels of circulating IGF-1 (geometric mean [95% CI]: 120 [38,378] µg/L).

**Conclusions:**

IGF-1 serum levels are significantly lower among women with the CC genotype for *IGF-1*-rs1520220. High soy isoflavone intake may interact with carrying CC genotype for *IGF-1*-rs1520220 to lower women's serum IGF-1 levels more.

## Introduction

The insulin-like growth factor (IGF) -system mainly consists of IGF-1, IGF-2, IGF receptors (IGF-1R and IGF-2R), and six binding proteins (IGFBP-1-6). Within IGF system, IGF-1 and its main binding protein, IGFBP-3, are two key molecules for cellular proliferation, differentiation and apoptosis [1]. In addition to the regulation of normal cell growth, IGF systems have been implicated in carcinogenesis [2]. Results from in vivo carcinogenesis models and epidemiological studies indicate that high levels of circulating IGF-1 are associated with increased risk and progression of several common cancers, including breast [3]–[7], prostate [8]–[11], colorectal [12]–[15], and ovarian cancer [16]–[17] among others. The relationship between circulating IGFBP-3 levels and cancers have been inconsistent [14], [16], [18]–[22].

Circulating IGF-1 and IGFBP-3 levels are determined by both heritable and exogenous factors. Two twin studies estimated that approximately 40%–60% of inter-individual differences in circulating levels of IGF-1 and IGFBP-3 are attributed to heritable factors, such as single nucleotide polymorphisms (SNPs) [23]–[24]. Although there are many SNPs identified for IGF-1 (rs1520220, rs10735380, rs5742665, rs1549593, rs2373722, etc.) and IGFBP-3 genes (rs2854744, rs2854746, rs3110697, rs2132570, rs2270628, etc.) [25], SNP rs1520220, located in the third intron of IGF-1 gene, and rs2854744, in the promoter region of the IGFBP3 gene, are consistently found to be associated with circulating IGF-1 and IGFBP-3 levels in Caucasians[26]–[29]. However, the associations of both SNPs with circulating IGF-1 and IGFBP-3 levels is not clear in the Chinese population as few studies have focused on this population. Exogenous factors, including both hormonal and nutritional, may have an important influence on circulating IGF-1 and IGFBP-3 levels [30]. Decreased levels of serum IGF-1 and IGFBP-3 were observed after chronic or acute energy restriction [31]. Several cross-sectional studies have found that serum IGF-1 concentrations are positively associated with higher total protein intake [32]–[34]. Soy isoflavone, a phytoestrogen, may act as selective ER modulators do by inhibiting IGF-1 concentrations or IGF system signaling [35].

In our previous studies, we mainly focused on breast cancer etiology and to be consistent with other studies in finding that nutrients, for example animal protein [36]–[37] may increase breast cancer risk, while high intake of soy products [38]–[39] may result in risk reduction. Given the associations of SNPs and dietary intake with circulating levels within the IGF system (IGF-1, IGFBP-3 and the molar ratio of IGF-1 to IGFBP-3), the hypothesis that genetic and nutritional factors may interact to effect serum concentrations of the IGF system and subsequent cancer risk warrants further investigation [30]. Several studies conducted among Caucasian women have examined the effects of nutritional factors and *IGF* polymorphisms on IGF serum levels [26], [28], [30], [33]–[34]. However, to the best of our knowledge, no previous study has examined this issue in Chinese population in China, and previous studies did not focus on the interaction of genetic and environmental factors. In the present study, we examined the main effects of dietary intake and genetic polymorphisms on circulating IGF-1 and IGFBP-3 concentrations among women aged 40 years or older, a population with high breast cancer incidence in China (more than 42.3 per 100,000) [40]. Furthermore, we explored potential combined effects of dietary factors and gene polymorphisms on IGF-1 and IGFBP-3 concentrations.

## Methods

### Ethics Statement

The study protocol was approved by the Institutional Review Board at Sichuan University. All subjects provided written informed consent before completing the questionnaire survey and laboratory tests.

### Study population

From September 2011 to July 2012, a total of 279 women aged 40 years and older sought the outpatient service for a physical examination from the Comprehensive Guidance Center of Women's Health, Chengdu Women's and Children's Central Hospital. The women who were of Han ethnicity, had been living in Sichuan Province for over 20 years, had no history of bilateral ovariectomy, no hormonal contraceptive use, and no perimenopausal complaints. We further excluded women who declined to join the program, women with insulin-dependent diabetes mellitus, and women with diagnosed/history of malignancy, including breast, liver or ovarian cancer at baseline as these diseases may affect circulating IGF levels. Ultimately, 143 women were included in the study. To minimize possible impact of estrogens from periodical variation during the menstrual cycle on IGFs, all women were enrolled between the 3^rd^ and 5^th^ day from the beginning of their periods for blood sample collection.

### Data collection

Information on socio-demographic and reproductive characteristics was collected using a structured questionnaire. A semi-quantitative dietary questionnaire, the Questionnaire of Health Related Dietary Habits, was designed to collect all participants' long-term (≥ 5 years) dietary habits. Evaluation of reliability and structural validity for the questionnaire has been described in detail in our previous study [39]. In brief, we first calculated the total daily intake of energy, protein, fat, carbohydrate, dietary fiber, and soy isoflavones referring to the nutrient compositions listed in the Danone Institute China Diet Nutrition Evaluating System [41]. We subsequently calculated the daily intake of energy-adjusted dietary factors to prevent potential underreporting of dietary intake using residual methods [39]. According to Chinese Dietary Reference Intakes (DRI) (Chinese DRIs committee formulated in 2000) for 18–50 year old women with moderate physical labor [42], we used the following dichotimization for total daily energy (2300 kcal/day), protein (70 g/day) and fat intake (77 g/day). For those without recommended levels of dietary intake, the means of dietary intakes (167.0 g/day for carbohydrate, 19.8 g/day for dietary fiber and 11.6 mg/day for soy isoflavones) were selected as cut-off values of high/low intake.

### Genotype analyses

Whole blood samples (5 mL) were obtained from participants via venipuncture into tubes containing ethylenediaminetetraacetic acid. Samples were stored at −20°C until DNA extraction. Genomic DNA was extracted from whole blood using TIANamp Blood DNA Kit (TIANGEN, Beijing). *IGF-1* rs1520220 and *IGFBP-3* rs2854744 were genotyped with TaqMan assays which were purchased from ABI (Applied Biosystems, Foster City, CA). All TaqMan assays were performed with the ABI 7500 thermal cycler (Applied Biosystems, Foster City, CA). In addition, duplicate detection was performed for 10 random samples (6% of the total subjects), and the observed concordance rate was 100%.

### Serum proteins measurement

A separate 3 mL of whole blood was withdrawn and then transported immediately to the laboratory, where samples stood for 2 hours, and then spun at 2500 g for 15 min, after which the serum was extracted and stored at −70°C until analysis.

Circulating total IGF-1 and IGFBP-3 levels were measured using enzyme-linked immunosorbent assay kits (Diagnostic Systems Laboratories, Webster, TX) according to manufacturers' instructions. Duplicate aliquots from each blood sample were analyzed for each individual, and the average of the two measurements was used for data analyses. Coefficients of variation (CVs) for duplication were less than 10%. The intra- and inter-assay coefficients of variation were 4.1% and 12.8%, respectively, for IGF-1 at a concentration of 110 ng/ml. Intra and inter-assay coefficients of variation were 4.9% and 5.4%, respectively, for IGFBP-3 at a concentration of 4,900 ng/ml. **The molar ratio, which may estimate the biologically active fraction of IGF-1, was calculated based on equation 1:**

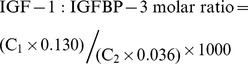



C_1_ and C_2_ are concentrations of IGF-1 and IGFBP-3, respectively. For IGF-1, 1 ng/ml is equal to 0.130 nM, and for IGFBP-3, 1 ng/ml is equal to 0.036 nM.

To ensure blinded laboratory analyses, participants were assigned a unique random ID number at each clinic visit.

### Statistical analyses

We checked the Hardy–Weinberg equilibrium (HWE) among all women via Chi-square test. The measured total IGF-1 and IGFBP-3 levels were log transformed to reduce departures from the normal distribution and then described using the geometric mean (95% confidence interval [CI]). After making a scatter plot of IGF component levels (i.e. IGF-1, IGFBP-3, and the molar ratio of IGF-1 to IGFBP-3) by age, we found that IGF levels were relatively more centralized among women aged less than 50 years. Considering differences in sex steroid levels between two age groups, which may affect circulating IGF levels, we divided the participants into two subgroups for sub-group analyses: <50 years and ≥50 years. Independent-sample T-tests and Chi-square tests/Fisher's exact tests were used to compare the demographic characteristics, and reproductive and dietary factors between age subgroups. Independent-sample T-tests were used to compare geometric means of IGF-1, IGFBP-3, and the molar ratio of IGF-1 to IGFBP-3 (equation 1), for each exposure of interest. Factors that were significantly associated with IGF component levels were then adjusted for as potential confounders in the covariance analyses to test the differences of IGF component levels within gene-diet exposure groups. Referring to results from previous studies that noted carrying the CC genotype for *IGF-1* rs1520220 was associated with lower IGF-1 levels in breast tissue among Chinese women [43], and that circulating levels of IGFBP-3 were significantly lower for CC or CA genotype carriers for *IGFBP-3* rs2854744 than AA allele carriers [44], we analyzed the effects within the recessive model for *IGF-1* rs1520220 (CC vs. GC+GG) and *IGFBP-3* rs2854744 mutant allele (AA vs. CC+CA) in IGF component levels.

The data were input into a database created with Epidata3.1 and analyzed with SPSS18.0 software.

## Results

### General demographic characteristics, dietary intake, and related reproductive factors

The demographic characteristics, reproductive and dietary factors, and insulin-like growth factor levels of our population were compared. We found no significant difference of these factors between age subgroups of <50 years and ≥50 years (Table 1).

**Table 1:** Descriptive statistics of demographic, reproductive and insulin-like growth factors by age status.

Variables	<50 years		≥50 years		*P* value^d^
	N	statistical distribution	N	statistical distribution	
BMI(kg/m^2^)^ b^	103	22.2±2.5	38	22.4±2.6	0.72
WHR^ b^	89	0.8±0.06	35	0.8±0.05	0.21
Age at menarche (years)^ b^	103	13.5±1.4	37	13.9±1.5	0.08
Passive smoking, yes, n (%)	69	68.3	25	71.4	0.73
Parity, ≥2, n (%)	16	16.5	7	19.4	0.69
Breast feeding, ≥3 months, n (%)	75	75.0	22	71.0	0.66
Total energy intake (kcal/day)^ b^	104	1566.4±645.3	39	1520.1±421.6	0.68
Protein intake (g/day)^a, b^	104	64.1±17.1	39	67.4±10.5	0.17
Fat intake (g/day)^a, b^	104	71.0±25.9	39	66.5±19.6	0.33
Carbohydrate intake (g/day)^a, b^	104	165.2±49.0	39	171.7±41.2	0.47
Dietary fiber intake(g/day)^a, b^	104	19.5±7.4	39	20.8±5.2	0.30
Soy isoflavone intake (mg/day)^a, b^	104	12.4±17.7	39	9.5±8.3	0.32
Insulin-like growth factor-1 (µg/L)^c^	104	168.0 (36.2, 779.0)	39	200.3 (35.7, 1125.3)	0.25
Insulin-like growth factor binding protein-3 (µg/L)^ c^	104	1837.5 (594.6, 5678.9)	39	1669.2 (508.7, 5476.6)	0.38
IGF-1:IGFBP-3 molar ratio^ c^	104	330.2(63.5,1716)	39	433.3(76.6,2451.6)	0.09

a : The dietary key nutrient intakes, including protein, fat, carbohydrates, dietary fiber, and soy isoflavones are adjusted for energy by residual method; ^b^: mean ± standard deviation;. ^c^: Geometric mean (95% confidence internal); ^d^: *P* value is based on T test for continuous variables and χ^2^ test or Fisher's exact test for categorical variables. Waist to hip ratio, WHR; Body mass index, BMI.

Geometric mean (95% CI) of IGF-1 and IGFBP-3, and the molar ratios for the participants are shown in Table 2 and stratified by measures of dietary intake. Among all participants, IGF-1 levels were positively correlated to both IGFBP-3 and the molar ratio of IGF-1to IGFBP-3 (Spearman's correlation coefficient: *r* = 0.44 and *r* = 0.70, respectively; both *P*<0.05). Average daily intake of key nutrients among all study participants were as follows (mean level±standard deviation [SD]): 1553.8±591.7 kcal/day for energy intake, 65.0±15.6 g/day for energy-adjusted protein, 69.8±24.4 g/day for fat, 167.0±46.9 g/day for carbohydrates, 19.8±6.9 g/day for dietary fiber, and 11.6±15.8 mg/day for soy isoflavones. While protein was not associated with any IGF component among all women, we found borderline significantly higher levels of IGFBP-3 among women with high protein intake compared to women classified as having low intake (*P* = 0.06) in the <50 age group. We also found a higher molar ratio of IGF-1 and IGFBP-3 for participants with high dietary fiber consumption (*P* = 0.03) among women ≥50 years. Total energy intake, daily intake of energy-adjusted fat, carbohydrate, and soy isoflavone were not associated with serum levels of IGF components.

**Table 2:** Relationship between geometric mean (95% CI) of insulin-like growth factor component levels with dietary intake.

Variables		N	IGF-1 (µg/L)	IGFBP-3 (µg/L)	IGF-1:IGFBP-3
**Overall**					
Total energy intake (kcal/day)	<2300	130	181 (36,906)	1782(580,5476)	367 (67,2015)
	≥2300	13	134(36,498)	1874(469,7492)	258(66,1009)
	*P* value		0.20	0.77	0.16
Protein intake (g/day)	<70	93	178(35,913)	1694(525,5467)	380(67,2161)
	≥70	50	173 (38,790)	1984 (679,5796)	315 (66,1499)
	*P* value		0.85	0.12	0.22
Fat intake (g/day)	<77	100	182 (34,981)	1834 (585,5746)	359(65,1998)
	≥77	43	163 (42,633)	1692(534,5362)	348 (69,1757)
	*P* value		0.45	0.45	0.85
Carbohydrate intake (g/day)	<167.0	70	163(35,756)	1747(577,5286)	337(64,1779)
	≥167.0	73	190 (37,978)	1833(561,5986)	375(68,2060)
	*P* value		0.25	0.62	0.46
Dietary fiber intake(g/day)	<19.8	67	155(37,647)	1793 (548,5873)	312 (61,1608)
	≥19.8	76	197 (36,1078)	1787 (587,5439)	399(73,2180)
	*P* value		0.07	0.97	0.09
Soy isoflavone intake (mg/day)	<11.6	90	193(36,1022)	1870 (541,6465)	372 (63,2193)
	≥11.6	53	151 (37,622)	1662(642,4303)	329(72,1504)
	*P* value		0.09	0.21	0.39
**<50 years**					
Total energy intake (kcal/day)	<2300	92	173 (37,816)	1840(617,5485)	340(64,1814)
	≥2300	12	133 (34,526)	1821(435,7620)	264(64,1086)
	*P* value		0.28	0.95	0.33
Protein intake (g/day)	<70	68	163(38,711)	1700(542,5334)	346 (63,1917)
	≥70	36	178(34,934)	2129 (742,6111)	302(65,1392)
	*P* value		0.59	**0.06**	0.43
Fat intake (g/day)	<77	68	176(34,905)	1924 (623,5944)	330 (59,1844)
	≥77	36	154(41,577)	1684 (547,5188)	330(72,1521)
	*P* value		0.41	0.26	0.99
Carbohydrate intake (g/day)	<167.0	55	158(34,724)	1774 (581,5421)	321 (67,1552)
	≥167.0	49	180 (38,849)	1911 (607,6022)	341(60,1942)
	*P* value		0.39	0.51	0.72
Dietary fiber intake(g/day)	<19.8	54	153 (35,667)	1734(508,5917)	319 (65,1575)
	≥19.8	50	186(38,911)	1957(714,5363)	342 (62,1905)
	*P* value		0.22	0.29	0.68
Soy isoflavone intake (mg/day)	<11.6	63	184 (38,889)	1934(567,6600)	344(64,1847)
	≥11.6	41	146 (34,620)	1699(657,4393)	311(62,1551)
	*P* value		0.14	0.26	0.55
**≥50 years**					
Total energy intake (kcal/day)	<2300	38	202 (35,1158)	1649(499,5445)	443(78,2512)
	≥2300	1	140	2650	191
	*P* value		–	–	–
Protein intake (g/day)	<70	25	226(32,1618)	1677 (471,5971)	486 (84,2821)
	≥70	14	162(53,496)	1655 (567,4835)	353(66,1879)
	*P* value		0.19	0.95	0.28
Fat intake (g/day)	<77	32	196 (33,1180)	1656 (517,5305)	428(79,2310)
	≥77	7	220(50,968)	1729 (431,6945)	459(58,3662)
	*P* value		0.76	0.87	0.85
Carbohydrate intake (g/day)	<167.0	15	183(36,917)	1648 (548,4962)	400(55,2905)
	≥167.0	24	212 (35,1306)	1682 (476,5943)	456(93,2244)
	*P* value		0.61	0.92	0.66
Dietary fiber intake(g/day)	<19.8	13	162 (45,590)	2063 (765,5565)	284(44,1838)
	≥19.8	26	223(33,1482)	1501 (434,5190)	535 (116,2483)
	*P* value		0.30	0.12	**0.03**
Soy isoflavone intake (mg/day)	<11.6	27	215(33,1411)	1730(481,6219)	449(64,3170)
	≥11.6	12	171(45,647)	1541(576,4120)	401(128,1255)
	*P* value		0.46	0.59	0.66

Insulin-like Growth Factor, IGF; Insulin-like Growth Factor Binding Protein, IGFBP; *P* value is based on T test.

Circulating levels of IGF components, stratified by demographic and reproductive characteristics, are shown in Table S1. Among women <50 years, we observed weak negative associations between WHR and circulating IGF-1 (*P* = 0.06). Passive smoking was associated with higher circulating levels of IGF-1 with borderline significance (*P* = 0.056). Age at menarche was positively associated with IGF-1 levlels among women≥50 years (*P* = 0.003). While associations between active smoking, drinking, and IGF components were of interest, small cell sizes (*N* = 2 and *N* = 4 for smoking and drinking, respectively) limited our ability to test these associations.

### *IGF-1* rs1520220/*IGFBP-3* rs2854744 genotypes and circulating IGF component levels

Among all women, the frequency of the C allele for *IGF-1* rs1520220 and the C allele for *IGFBP-3* rs2854744 were 56.6% and 27.1%, respectively. Genotypes of *IGF-1* rs1520220 and *IGFBP-3* rs2854744 did not deviate from HWE (*IGF-1* rs1520220: *χ^2^* = 3.04, *P* = 0.08; *IGFBP-3* rs2854744: *χ^2^* = 2.29, *P* = 0.13). Table 3 presents covariance analyses results of *IGF-1* rs1520220 and *IGFBP-3* rs2854744 genotypes in relation to circulating levels of IGF components. Among women <50 years, circulating IGF-1 levels were significantly lower for those carrying the CC genotype for *IGF-1* rs1520220 than IGF-1 levels for those carrying GC or GG genotypes (recessive model: *P* = 0.007). We did not observe any association between *IGFBP-3* rs2854744 genotypes and circulating IGFBP-3 level or the molar ratio.

**Table 3:** Relationship between geometric mean (95% CI) of insulin-like growth factor component levels with IGF-1 and IGFBP-3 genotypes.

Variables		N	IGF-1 (µg/L)	IGFBP-3 (µg/L)	IGF-1:IGFBP-3
**Overall**					
IGF-1	GG+ CG	92	191 (35,1039)	1819(574,5765)	380(68,2115)
	CC	51	152(39,589)	1738(558,5412)	316(63,1576)
	*P* value		0.10	0.66	0.22
IGFBP-3	CC+CA	63	161(35,731)	1723(566,5251)	337(57,2009)
	AA	79	187 (36,963)	1838(567,5957)	368(74,1831)
	*P* value		0.23	0.70	0.57
**<50 years** a					
IGF-1	GG+ CG	66	180(36,8980)	1907(634,5731)	341(61,1900)
	CC	38	149 (37,597)	1724(530,5605)	313(67,1450)
	*P* value		**0.007**	0.16	0.46
IGFBP-3	CC+CA	45	164 (34,800)	1759(558,5546)	337(55,2075)
	AA	59	171 (38,773)	1900(621,5812)	325 (71,1489)
	*P* value		0.76	0.44	0.80
**≥50 years^b^**					
IGF-1	GG+ CG	26	224(34,1478)	1616(454,5754)	501(99,2534)
	CC	13	160 (44,591)	1781(629,5044)	325(50,2116)
	*P* value		0.29	0.97	0.25
IGFBP-3	CC+CA	18	153(39,597)	1638(575,4670)	337 (59,1945)
	AA	20	244(36,1667)	1666(434,6396)	528(99,2823)
	*P* value		0.14	0.85	0.25

Insulin-like Growth Factor, IGF; Insulin-like Growth Factor Binding Protein, IGFBP.

a : Models among women <50 years were adjusted for WHR (waist hip ratio), passive smoking and protein intake; ^b^: Models among women ≥50 years were adjusted for age at menarche and dietary fiber intake.

### Combination of *IGF-1* rs1520220/*IGFBP-3* rs2854744 genotypes and daily intake of energy-adjusted soy isoflavones and protein on circulating IGF component levels among women <50 years old

For women <50 years, thosewith both low intake of soy isoflavones and at least one variant G allele for *IGF-1* rs1520220 had the highest levels of circulating IGF-1 (geometric mean [95% CI]: 195 [37, 1021] µg/L). Women who either had high intake of dietary soy or were homozygous for the major C allele had significantly lower levels of circulating IGF-1 (geometric mean [95% CI]: 160 [34, 753] µg/L and 167 [38, 723]µg/L, respectively). Women who had both high soy intake and were homozygous for the major C allele had the lowest levels of circulating IGF-1 (geometric mean [95% CI]: 120 [38, 378] µg/L) (Table 4, *P* = 0.002). As for combination of *IGFBP-3* rs2854744 genotypes and soy isoflavone intake, we found higher IGFBP-3 levels inwomen with low soy intake (groups 1 and 2: (geometric mean [95% CI]: 1832 [503, 6671] µg/L and 2014 [613, 6613] µg/L, respectively)) than those women with high soy intake (groups 3 and 4: (geometric mean [95% CI]: 1654 [663, 4124] µg/L and 1735 [641, 4696] µg/L, respectively)), but these differences appear to be driven primarily by soy intake, as the overall effect of diet and genetics was not significant (*P = *0.40). Although protein intake were found to be related to IGF system variables (Table 2), combinations of *IGF-1* rs1520220/*IGFBP-3* rs2854744 genotypes and daily intake of energy-adjusted protein were not associated with IGF component levels (*P*>0.05).

**Table 4:** Relationship between geometric mean (95% CI) of insulin-like growth factor component levels with IGF-1 and IGFBP-3 genotypes combined soy isoflavone and protein intake among women <50 years old.

Group	Dietary intake	IGF-1 genotypes	N	IGF-1(µg/L)	IGFBP-3(µg/L)	IGF-1: IGFBP-3	IGFBP-3 genotypes	N	IGF-1(µg/L)	IGFBP-3(µg/L)	IGF-1: IGFBP-3
**Soy isoflavone intake**											
1	low (<11.6)	GG+ CG	38	195 (37,1021)	2067(617,6916)	342(60,1932)	CC+CA	27	174(39,776)	1832(503,6671)	344(64,1832)
2	low (<11.6)	CC	25	167(38,723)	1747(497,6132)	345(67,1779)	AA	36	191(36,997)	2014(613,6613)	343(61,1901)
3	high (≥11.6)	GG+ CG	28	160(34,753)	1708(685,4257)	338.9(60,1917)	CC+CA	18	149(26,850)	1654(663,4124)	326(41,2576)
4	high (≥11.6)	CC	13	120(38,378)	1678.8(580,4862)	257.3(71,931)	AA	23	144(43,480)	1735(641,4696)	299 (92,972)
	*P* value a			**0.002**	0.13	0.61			0.37	0.40	0.95
**Protein intake**											
1	low	GG+ CG	44	176 (38,830)	1780(5780,5477)	358(61,2109)	CC+CA	31	159 (32,802)	1595(485,5240)	361(53,2461)
2	low	CC	24	141 (38,522)	1562(478,5107)	326 (65,1649)	AA	37	166 (42,650)	1793(591,5436)	335(72,1560)
3	high (≥70)	GG+ CG	22	187(32,1083) (32,1083)	2187(786,6091)	309 (61,1569)	CC+CA	14	175(37,836)	2183(836,5696)	289(59,1425)
4	high (≥70)	CC	14	164(35,769)	2041 (657,6346)	290 (70,1203)	AA	22	180(31,1037)	2096(676,6499)	310(68,1419)
	*P* value a			0.30	0.09	0.61			0.96	0.14	0.65

Insulin-like Growth Factor, IGF; Insulin-like Growth Factor Binding Protein, IGFBP.

a : Models among women <50 years were adjusted for WHR (waist hip ratio), passive smoking and protein intake.

While dietary fiber intake seems to be positively related to the molar ratio of IGF-1 to IGFBP-3 in the ≥50 age group (Table 2), we did not analyze combinations of *IGF-1* rs1520220/*IGFBP-3* rs2854744 genotypes and dietary fiber intake due to limited sample size in this age group.

## Discussion

In this study, we aimed to reveal the genetic and dietary determinants of IGF-1 and IGFBP-3 in our population of Chinese women aged 40 years or older. We found that carrying CC genotype for IGF-1 rs1520220 may work alone or interact with high soy food intake to decrease serum IGF-1 levels among women <50 years old.

Serum concentrations of IGF-1 and IGFBP-3 are mainly determined by genetic factors [23]-[24]. Although SNP *rs1520220* is located in the intron of *IGF-1* gene, it may influence circulating IGF-1 expression via altering the secondary structure of RNA[45] or DNA [46]. In this study, we observed that IGF-1 levels for carriers of CC genotype for *IGF-1* rs1520220 were significantly lower than that for G allele carriers. In another study, carrying CC genotype for *IGF-1* rs1520220 was associated with lower IGF-1 levels in breast tissue among 403 Chinese women [43], which is similar to results of our study. Guet al. 2010 produced consistent results from 5533 women and 5379 men that carrying the G allele for *IGF-1* rs1520220 was associated with increased IGF-1 levels [29]. However, a previous study with 345 women from the United Kingdom also examined the association between *IGF-1* tagging polymorphisms and circulating IGF-1 levels, and found that the C allele for *IGF-1* rs1520220 was associated with increased circulating IGF-1 in 345 females [26]. The C to A mutation for *IGFBP-3* rs2854744 may theoretically result in reduced promoter activity, and in turn, decreased levels of circulating IGFBP-3 [28]. This SNP has been found to be strongly associated with IGFBP-3 levels in several studies among Caucasian women [26]-[28]; however, we did not observe this association in our study. Our lack of association may be due to the low frequency of the C allele in our study population (27.1%) compared to Caucasian populations (∼53.0% in women) [47]-[48]. However, the observed frequency of the C allele among women living in the Sichuan province is similar to the frequency reported in a large population-based study in Shanghai, China (23.0%) [49]. Therefore, we believe that any discrepancies observed between our study and those conducted among Caucasian women may be attributed to racial diversity. In addition to genetic polymorphisms, dietary factors may influence circulating IGF system levels in humans. Several mechanisms have been proposed [30]: i. Approximately 80% of circulating IGF-1 are synthesized in the liver and exogenous factors, such as diet, may directly influence hepatic IGF-1 expression, synthesis, and secretion, resulting in altered serum IGF levels; ii. Certain dietary factors (i.e. protein and fat intake) could affect IGFBP levels, and may also influence the binding ability of IGF-1 to IGF-1R by competing for active sites; iii. Dietary factors may indirectly affect the IGF system through interaction with genetic factors.

Soy food has consistently been characterized to have an anticancer effect [50], particularly in Asian populations where soy intake is relatively high. *In vitro* studies have shown that pharmacological doses of genistein, the main composition of soy isoflavones, may stimulate IGF-1R signaling human breast cancer cells [51].Although we did not find associations between soy intake and serum IGF-1 as previous studies did [52], we observed that high soy intake enhanced the association between carrying CC genotype for *IGF-1* rs1520220 and lower circulating IGF-1 levels among women aged <50 years. Since relatively high circulating IGF-1 concentration is associated with increased cancer risk [18]-[19], modulation of IGF-1 levels by soy isoflavone intake may be implemented as a risk reduction mechanism, particularly for breast cancer as reported in our previous studies [38]-[39]. We also hypothesized that *IGFBP-3* polymorphisms may potentially interact with dietary intake, for example, soy isoflavone intake, and then influence circulating IGF component levels. However, we found negative results, which may be due partly to the limited sample in our study. This hypothesis deserves further study with a larger sample size.

Total energy and dietary protein have been shown to be positively associated with serum IGF levels [32]–[34]. We observed no associations between total energy and IGF component levels, although there was a borderline significant positive association for dietary protein and IGFBP-3, it was limited to women aged <50 years (Table 2). In this age group, positive results disappeared when we combined protein intake and *IGF-1* rs1520220/*IGFBP-3* rs2854744 genotypes. Few studies paid attention to the relationship of dietary fiber intake and circulating IGF levels. Gannet al. 2005 found low fat, high fiber dietary intake didn't change circulatory IGF-1 or IGFBP-3 levels compared to the usual diet among women in Chicago after a intervention period of 12 menstrual cycles [53]. However, the main effect of dietary fiber intake isn't clear yet. In our study, while we observed a possible relationship of high dietary fiber intake with increased molar ratio of IGF-1 to IGFBP-3 in the ≥50 age group, relativaely limited sample (*N* = 39) may have limited our ability to ascertain the association of dietary fiber intake combined with *IGF-1* rs1520220/*IGFBP-3* rs2854744 genotypes with IGF component levels. We believe that the effects of fiber intake on IGF system levels and corresponding cancer risk deserve further study.To our knowledge, our study is the first to explore the combined- influence of *IGF-1* rs1520220/IGFBP-3 rs2854744 and dietary factors on IGF component levels. Although it is a cross-sectional study among hospital-based women, with limited sample size, we only selected the women who sought a physical examination and not those who suffering from any disease that may affect circulating IGF levels; we also implemented strict quality control measures throughout the study, including data collection, laboratory tests and statistical analyses. Thus, we believe our findings reflect the correlation between *IGF-1* rs1520220/IGFBP-3 rs2854744, dietary intake, and IGF levels in the general population of Chinese women aged 40 years and older. In addition, the observed combined influence of soy isoflavone consumption and *IGF-1* rs1520220 genotypes on IGF-1levels may reflect some underlying biological interaction of the *IGF-1* gene and diet on serum IGF component levels. This requires confirmation in larger prospective studies. A randomized control trial of soy isoflavone supplements would aid our understanding of these interactions and help expose the anticancer mechanism of soy isoflavones.

## Supporting Information

Table S1 Associations between general demographic, reproductive factors and circulating IGF components levels.
(DOCX)
