# Supplementary material for: Genetic and Dietary Determinants of Insulin-Like Growth Factor (IGF)-1 and IGF Binding Protein (BP)-3 Levels among Chinese Women
Source: PLoS One. 2014 Oct 6;9(10):e108934. doi: 10.1371/journal.pone.0108934 (PMC4186782; doi:10.1371/journal.pone.0108934)
Supplement: Table S1 — Associations between general demographic, reproductive factors and circulating IGF components levels. (DOCX) [file pone.0108934.s001.docx]

Table S1 Associations between general demographic, reproductive factors and circulating IGF components levels

| Variables | | N | IGF-1 (µg/L) | IGFBP-3 (µg/L) | IGF-1：IGFBP-3 |
| --- | --- | --- | --- | --- | --- |
| Overall | | | | | |
| BMI (kg/m^2^) | ≤24 | 107 | 179(37,870) | 1850(587,5829) | 349(69,1766) |
|  | >24 | 34 | 175(33,917) | 1635(529, 5053) | 386(574,2590) |
|  | *P* value |  | 0.89 | 0.29 | 0.56 |
| WHR | ≤0.8 | 57 | 196 (36,1063) | 1785 (591,5395) | 397(81,1933) |
|  | >0.8 | 67 | 172 (35,862) | 1746(519,5867) | 357(57,2236) |
|  | *P* value |  | 0.40 | 0.83 | 0.50 |
| Passive smoking | No | 42 | 153(50,467) | 1782 (584,5442) | 310(66,1463) |
|  | Yes | 94 | 186 (32,1094) | 1834(583,5769) | 367(64,2111) |
|  | *P* value |  | 0.13 | 0.79 | 0.29 |
| Age at menarche (years) | ≤12 | 27 | 195(39,986) | 2145(777,5920) | 328 (53,2030) |
|  | >12 | 113 | 174(35,860) | 1716(536,5496) | 365 (69,1938) |
|  | *P* value |  | 0.51 | 0.08 | 0.57 |
| Parity | 1 | 110 | 185(36,960) | 1873(611,5741) | 356(63,2019) |
|  | ≥2 | 23 | 171(40,741) | 1517(410,5609) | 406 (77,2154) |
|  | *P* value |  | 0.68 | 0.12 | 0.51 |
| Breast feeding (months) | <3 | 34 | 166(43,635) | 1917 (747,4923) | 312 (77,1270) |
|  | ≥3 | 97 | 179 (33,967) | 1804(558,5837) | 359(60,2137) |
|  | *P* value |  | 0.63 | 0.59 | 0.37 |
| <50 years | | | | | |
| BMI (kg/m^2^) | ≤24 | 79 | 167(39,713) | 1895(612,5870) | 317 (68,1474) |
|  | >24 | 24 | 176 (28,1092) | 1641 (528,5101) | 388(53,2831) |
|  | *P* value |  | 0.76 | 0.29 | 0.31 |
| WHR | ≤0.8 | 43 | 210(39,1144) | 1929(641,5805) | 393(95,1631) |
|  | >0.8 | 46 | 151(34,674) | 1698 (521,5534) | 321(48,2138) |
|  | *P* value |  | 0.06 | 0.31 | 0.27 |
| Passive smoking | No | 32 | 138(51,374) | 1766 (552,5657) | 281 (63,1262) |
|  | Yes | 69 | 180(33,974) | 1851(597,5740) | 350 (64,1927) |
|  | *P* value |  | 0.056 | 0.71 | 0.22 |
| Age at menarche (years) | ≤12 | 23 | 212(39,1163) | 2168 (826,5690) | 353 (53,2351) |
|  | >12 | 80 | 158(36,691) | 1746(545,5592) | 327(67,1593) |
|  | *P* value |  | 0.11 | 0.11 | 0.70 |
| Parity | 1 | 81 | 180 (36,918) | 1948 (646,5872) | 335(60,1860) |
|  | ≥2 | 16 | 145(50,418) | 1456 (414,5123) | 359 (78,1651) |
|  | *P* value |  | 0.31 | 0.07 | 0.76 |
| Breast feeding (months) | <3 | 25 | 147 (54,402) | 1890(716,4989) | 282(85,929) |
|  | ≥3 | 75 | 177(34,922) | 1853(593,5787) | 344 (59,2012) |
|  | *P* value |  | 0.21 | 0.88 | 0.22 |
| ≥50 years | | | | | |
| BMI (kg/m^2^) | ≤24 | 28 | 218(33,1422) | 1729(518,5773) | 455 (78,2670) |
|  | >24 | 10 | 171 (49,600) | 1620 (500,5248) | 382(64,2275) |
|  | *P* value |  | 0.47 | 0.78 | 0.60 |
| WHR | ≤0.8 | 14 | 159 (30,842) | 1409(509,3897) | 408 (52,3213) |
|  | >0.8 | 21 | 231(41,1311) | 1854 (506,6800) | 449(86,2337) |
|  | *P* value |  | 0.23 | 0.20 | 0.77 |
| Passive smoking | No | 10 | 214 (61,757) | 1834 (674,4997) | 422 (82,2167) |
|  | Yes | 25 | 206(28,1517) | 1787(534,5978) | 416 (63,2734) |
|  | *P* value |  | 0.89 | 0.91 | 0.97 |
| Age at menarche (years) | ≤12 | 4 | 121(85,171) | 2017(477,8536) | 216(68,689) |
|  | >12 | 33 | 218(35,1338) | 1645 (507,5343) | 477 (81,2822) |
|  | *P* value |  | 0.003 | 0.54 | 0.10 |
| Parity | 1 | 29 | 196 (35,1110) | 1677 (527,5342) | 422 (71,2502) |
|  | ≥2 | 7 | 248(32,1929) | 1665 (371,7485) | 537(75,3857) |
|  | *P* value |  | 0.55 | 0.98 | 0.54 |
| Breast feeding (months) | <3 | 9 | 230(33,1612) | 1995(801,4968) | 416(66,2606) |
|  | ≥3 | 22 | 188 (30,1177) | 1647 (449,6038) | 413(64,2662) |
|  | *P* value |  | 0.60 | 0.44 | 0.98 |
| Insulin-like Growth Factor, IGF; Insulin-like Growth Factor Binding Protein, IGFBP; Waist to hip ratio, WHR; Body mass index, BMI; *P* value is based on T test | | | | | |
